# The neural dynamics of political socio-pragmatic violations: an ERP study

**DOI:** 10.3389/fnhum.2026.1820376

**Published:** 2026-06-29

**Authors:** Manuel Hons, Edgar Onea, Ilse Schiller, Silvia Erika Kober

**Affiliations:** 1 Department of Psychology, University of Graz, Graz, Austria; 2 Department of German Studies, University of Graz, Graz, Austria

**Keywords:** contextual violations, ERP, N400, political discourse, pragmatics

## Abstract

**Introduction:**

Pragmatic violations are known to elicit reliable N400 modulations in event related potentials of electroencephalographic recordings. While this effect has been extensively documented in linguistic contexts, evidence from political discourse remains limited. To address this gap, the present study investigated neural responses to socio-pragmatic coherence in politically framed statements.

**Methods:**

Participants read political utterances containing critical target words that were either coherent or incoherent with a preceding description of the producer. To examine whether pragmatic processing could be altered by typographic cues, target words were presented either unmarked or enclosed in quotation marks.

**Results:**

Consistent with prior findings, pragmatically incoherent target words elicited a larger negative deflection peaking around 400 ms post-onset than coherent words. Notably, this pattern was reversed when target words were presented in quotation marks.

**Discussion:**

We interpret this reversal as evidence that quotation marks signal a rejection or distancing from a word’s conventional meaning, thereby altering contextual integration demands. While rejecting the meaning of incoherent terms (e.g., *“climate terrorists”*) may facilitate integration when aligned with the speaker’s stance, rejecting the meaning of coherent terms (e.g., *“climate activists”*) appears to increase processing difficulty. These findings demonstrate that typographic cues can modulate socio-pragmatic interpretation in political language, with measurable neural consequences.

## 1 Introduction

Pragmatics is a linguistic discipline concerned with the differentiation between what is said and what is implied, and integrates the context of use into the interpretation of speech (Recanati, 1989). The way in which language can refer to the context or the situation of an utterance, i.e., the linguistic deixis, is manifold. Deixis also concerns with how the interpretation of utterances is affected by certain contextual elements (Levinson, 1992). There are several important dimensions of deixis in linguistics: Apart from the person, time and place deixis, discourse and social deixis are considered crucial components to the language-context system (Levinson, 1992; Van Berkum, 2009). Neurolinguistic investigations are often interested in the latter two, especially with regards to pragmatic violations relating to the discourse or the social frame of utterances (Porkert et al., 2024; Van Berkum, 2009). Pragmatic violations are generally characterized by target stimuli violating expectations or constraints based on a previously defined context. In electroencephalographic (EEG) neurolinguistic research, pragmatic violations have been shown to elicit a characteristic event-related potential (ERP) pattern, most notably the N400 component (Kutas and Hillyard, 1980). This N400 effect is characterized as a more negative deflection in the ERP signal approximately 400 ms post-onset in response to contextually incoherent information and is most prominent along the centro-parietal midline (Brothers et al., 2020; Van Berkum et al., 1999; Van Berkum et al., 2003). The first observations of N400 arose from sentential incoherences, up until Kutas and Hillyard (St George et al., 1994) took a broader discourse context into account: Their participants were reading text which was only coherent when accompanied by a title. The authors then found increased N400 amplitudes in untitled versus titled paragraphs. The article by St George et al. (1994) further influenced the terminology used in the current paper, as it heavily relied on terms such as “coherent” and “incoherent” in relation to the context of a stimulus. What followed was an increase in neuro-pragmatic ERP studies extending their contextual focus to a broader discourse level (Federmeier and Kutas, 1999; Van Berkum et al., 2003); Van Berkum et al., 1999), for instance, reported said N400 effect on both a wider discourse and a sentence level. Whether the N400 effect is a result of a difficulty in integration or in memory retrieval is a matter of ongoing debate (Van Berkum, 2009). In both cases, the N400 amplitude is greater when anomalous, contextually incoherent stimuli are processed, indicating a larger consumption of cognitive resources (Lau et al., 2008). While numerous studies to date have replicated this N400 effect (Balconi and Caldiroli, 2011; Calma-Roddin and Drury, 2020; Dini et al., 2022; Filik and Leuthold, 2008; Kuo and Prat, 2024; Mahieux et al., 2024; Niedeggen et al., 1999; Weimer et al., 2019), equivalent research on specific violations occurring in political discourse remains limited.

In addition to textual and auditory linguistic contexts, various characteristics of both the speaker and the listener can also alter the processing of an utterance (Van Berkum, 2009): Existing literature suggests, for example, that individuals with diverse political orientations respond differently to political messages, particularly in terms of neural reactions (Leong et al., 2020; Mahieux et al., 2024; Van Berkum et al., 2009). Similarly, in neuro-pragmatic ERP research, a distinction is drawn between text-based, speaker-based and listener-based contexts (Van Berkum, 2009). In the following, all target words, i.e., those stimuli the ERP responses are locked to, are italicized. (Van Berkum, 2009) explored the ERP dynamics of devout Christians and non-religious individuals in response to moral statements, such as “I find euthanasia *acceptable*/*unacceptable*.”, with “*acceptable*” and “*unacceptable*” representing the target words to evoke an N400 effect. The authors found that value-inconsistent statements elicited a subtle N400 effect and a more pronounced late positive potential as compared to value-consistent statements. Consonantly, Mahieux et al. (2024) reported that, among American college students, Democratic voters showed a more pronounced N400 response when confronted with pro-Republican statements in contrast to pro-Democratic statements. In this example, Republican voters showed no such effect. Apart from recipient characteristics, subjective speaker stereotypes were also shown to evoke such N400 effects. One study, for instance, reported that when the sentence “If I only looked like *Britney Spears*.” was presented in a male voice, or when the sentence “I always drink a glass of *wine* before going to bed.” was presented in the voice of a child, an N400 effect was observed (Van Berkum et al., 2008). In another research study, socially inferred gender mismatches produced an N400 effect as well (Wu and Cai, 2025). An example for such an engendered social mismatch is the utterance “I’m going to have a *manicure* this weekend” spoken with a male voice. Interestingly, this social N400 effect decreased in extent as a function of the openness of the recipients. There are numerous prior studies indicating N400 effects caused by difficulties in integrating an utterance with the social context provided by implicit speaker characteristics, such as age or gender (Hanulíková and Carreiras, 2015; Kreiner et al., 2009; Van Berkum et al., 2008; Wu and Cai, 2025). Crucially, N400 effects are not exclusive to contextual violations: lexical properties, such as word frequency (Kutas and Federmeier, 2011), but also the emotional salience of words (Kanske and Kotz, 2007) have been shown to modulate N400 component, which makes controlling of potential confounds essential in neuro-pragmatic N400 research. While comparable studies often speak of the broader term of “pragmatics” (Jiang et al., 2013b; Pélissier and Ferragne, 2022; Van Berkum et al., 2008), we would like to provide a more specific positioning of the current study within the complex linguistic literary landscape: Despite there being no single unanimously agreed-upon definition of socio-pragmatics (Culpeper, 2021), we definitely see this research rooted in the field. Drawing from The Cambridge Handbook of Sociopragmatics (Culpeper, 2021), the field of socio-pragmatics is “focused on the construction and understanding of meanings arising from interactions between language […] and socio-cultural phenomena.” Further, it “considers norms emerging in such contexts, how they are exploited by participants, and how they lead to evaluations of (in) appropriateness.” An important distinction to the field of pragma-linguistics is to be made here, which is concerned with the linguistic manifestation of pragmatic force rather than social or socio-cultural phenomena (Culpeper, 2021).

Metacommunicative markers, such as quotation marks, can cause a change of context—a shift in focus, perspective and meaning by blocking the stereotypical interpretation of a quotation. Quotation marks are understood as minimal pragmatic markers—minimal, because they do not have an own semantic meaning and pragmatic, because contextual conclusions have to be drawn (Gutzmann and Stei, 2011). Citations, for instance, can cause a change in perspective; the citing instance does not necessarily adopt the views or statements expressed in the citation (Predelli, 2003). Apart from that, quotation marks can also signal an alternative meaning for a particular quote, e.g., “Our “discussion” ended with a broken nose.” indicates that the “discussion” was not actually a discussion but a physical fight, signaling ironic usage of the word (Schlechtweg and Härtl, 2023). Similarly, quotation marks can signal distancing or rejection of the stereotypical interpretation of a quotation—in political discourse, the expression “refugee,” for example, can signal rejection of the original meaning, implying that refugees are no refugees, i.e., not actually seeking refuge (Scharloth, 2021). We have found no studies investigating the effect of quotation marks on neural responses to pragmatic violations. But at least, there are studies that explore the influence of additional tasks during semantic processing. Hohlfeld et al. (2004a), Hohlfeld et al. (2004b), for instance, reported that additional processing cost delayed the N400 response during a language perception task. Hence, while it is currently unknown how quotation marks influence neural reactions specifically, there is a plausible generic expectation of a comparable N400 delay caused by quotation marks and the accompanying additional processing cost. Further, since quotation marks are able to signal distancing from an utterance, potential to alter the ERP pattern in response to textual stimuli can be seen here. To the best of our knowledge, no study exists that investigates this relationship.

Despite the fact that neurolinguistic paradigms designed to produce pragmatic violations primarily target the elicitation of N400 effects, there are additional ERP components that may be evoked: the P300 component (Van Berkum et al., 2009), characterized by a positive deflection after approximately 300 ms, and the late positive potential (LPP) (Kreiner et al., 2009; Van Berkum et al., 2009), described as a positive potential with an onset typically after 400 ms and a duration of a few hundred milliseconds, depending on the task (Fields, 2023). Modern theories on P300 and LPP components state that both are affected by stimulus significance, which in turn is mediated by stimulus frequency, emotional content, and stimulus relevance (Fields, 2023; Hajcak and Foti, 2020; Hajcak et al., 2010; Mühlberger et al., 2017). Thus, as indicated by previous research, variation in the subjective offensiveness of words might impact the expression of not only the P300 and the LPP, but also the N400 (Struiksma et al., 2022; Van Berkum et al., 2009). Historically, scientific literature has shown that both components were influenced by common variables, which is why it is argued that both might stem from the same underlying processes (Fields, 2023; Hajcak and Foti, 2020; Mühlberger et al., 2017). Consonantly, Van Berkum et al. (2009) showed that political value-inconsistent statements evoked not only an N400 component but also a P300 as well as a late positive potential (LPP).

In summary, context can appear manifoldly, with a variety of factors being capable of adjusting the probabilities of what needs to be expected next in an utterance. Consequently, there is also a myriad of contexts susceptible to contextual violations and accompanying N400 effects. Previous studies have shown that, apart from “classical,” text-based pragmatic violations, speaker as well as recipient characteristics, such as political attitudes and gender, are able to produce an N400 effect as well. When taking social information into account to evaluate utterances, we step into the field of socio-pragmatics. Literature indicates that metacommunicative markers, such as quotation marks, are also considered to produce contextual, interpretative shifts when it comes to processing speech or text. However, experimental studies exploring the pragmatic influence of those markers in a neurolinguistic manner remain limited. Finally, variations in emotional salience might lead to variations in P300 and LPP amplitude.

The present study aims to investigate the potential N400 effect related to text-based socio-pragmatic violations in political discourse using EEG. Statements of fictitious speakers are presented with target words either violating a given description of the speaker (contextually incoherent) or not (contextually coherent). Despite the notion that the term ‘political discourse’ can be variably interpreted, we consider our experimental design to capture a discourse scenario as the political statements are not to be interpreted in isolation but rather must be related to the previous person description. This is in line with the distinction between sentence and discourse level made by several other comparable neuro-pragmatic ERP literature (St George et al., 1994; Van Berkum, 2009; Van Berkum et al., 1999). It is anticipated that socio-pragmatic violations, i.e., contextually incoherent target words, will elicit a larger N400 amplitude compared to contextually coherent target words. Furthermore, the study explores whether the conventional N400 effect gets cancelled when target words appear enclosed in quotation marks. The rationale behind this assumption is that, due to the indication distancing oneself from the utterance, the anticipatedly more pronounced N400 amplitude in response to incoherent target words will be eliminated, resulting in a non-significant difference between coherent and non-coherent target words. Further, we explore whether a delay in the N400 elicitation occurs due to the additional processing step caused by the presentation of quotation marks. Finally, we anticipate that larger subjective offensiveness of target words elicits larger P300 and LPP amplitudes.

## 2 Materials and methods

### 2.1 Participants

For a within-subjects ANOVA comprising 4 measurements and a medium effect size (*f* = 0.25), power calculations based on G*Power (version 3.1.9.7.) (Faul et al., 2007) suggested a minimum sample size of 24. Furthermore, sample sizes of comparable studies were used to further gauge the prospective sample size: (Van Berkum et al., 2008) reported 24 participants, White et al. (2009) reported 23, and Van Berkum et al. (2009) reported 43. For the present study, data from 38 healthy individuals were used from a total sample of 48 participants. Seven datasets had to be excluded from the overall sample due to excessive artifacts in the EEG channels of interest and errors during data collection. Two datasets were excluded due to providing less than 15 usable data segments in at least one condition after our rigorous visual and semi-automatic artifact removal procedure. One dataset was excluded because the participant did not meet inclusion criteria. The remaining sample of 38 individuals consisted of 13 male participants and 25 female participants, aged 19 to 42 years (*M* = 27.58, SD = 5.24). All participants completed each condition of the study. General inclusion criteria for the study included a minimum age of 18 years, German as a first language, no psychiatric or neurological disorders, and no medication that could affect attention. Additionally, general EEG exclusion criteria were considered, including wounds, inflammation, and scars in the head area, as well as the presence of a pacemaker or brain stimulator. Participants were recruited through university forums, social media posts, and flyers. Participants received compensation in the form of €20.00 or study credits for their participation. Participants received written experiment information on a computer screen and provided informed consent by mouse click. The measurements were conducted from August 2024 to December 2024. All procedures of the present study have been conducted in accordance with the Declaration of Helsinki and were approved by the ethics committee of the University of Graz (GZ. 39/154/63 ex 2023/24). The procedures of this study were pre-registered at the Open Science Framework (OSF; link will be provided after blind peer review). Deviations from the pre-registration are listed in the Supplementary Table S1. The ERP data as well as the analysis code can be publicly accessed via the following link: https://osf.io/sgbqf.

### 2.2 Experimental material

#### 2.2.1 EEG equipment

**Figure 1:**
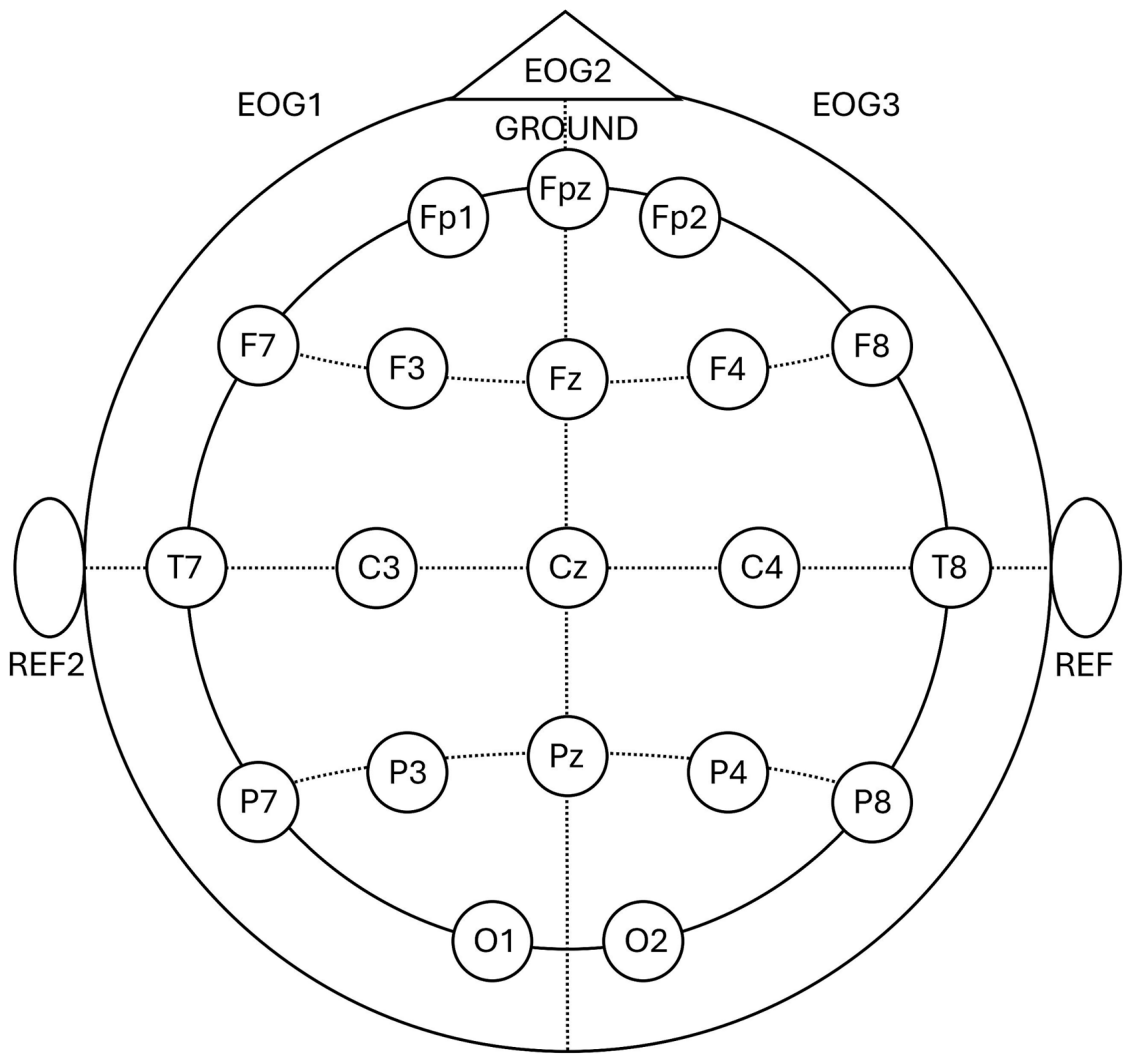
Electrode positions from which data were collected.

For the EEG measurements, 19 actiCAP active electrodes (Brain Products GmbH) were attached to the participants’ scalp according to the international 10–20 system (as shown in Figure 1). The BrainAmp EEG amplifier (Brain Products GmbH) was used. An electrooculogram (EOG) was measured via three electrodes: One to account for vertical eye movement and blinking, which was placed approximately 1 cm above the nasion and two further electrodes, which were placed at the lateral corners of the eyes to account for horizontal eye movement. The ground electrode was attached to the Fpz position. Two electrodes placed on the mastoids served as reference electrodes. The impedances of the reference electrodes and the ground electrode were kept below 10 kOhm. The impedances of the scalp and EOG electrodes were kept below 25 kOhm.

#### 2.2.2 Sociodemographic variables

At the beginning of each session, a standard assessment of sociodemographic variables was conducted, comprising age and gender, as these variables are potential influencing factors.

#### 2.2.3 Social dominance orientation

In the present study, participants’ Social Dominance Orientation (SDO) was assessed using the SDO-7 questionnaire (Ho et al., 2015; Pratto et al., 1994). SDO is a personality trait characterized by a preference for hierarchical intergroup relations and the belief that one’s in-group is superior to out-groups. It has been shown to correlate significantly with a range of ideological positions, including nationalism, anti-Black racism, and elitism, as well as with attitudes toward homosexual rights, social welfare programs, and environmental policies (Pratto et al., 1994). Accordingly, SDO was employed in this study as an indicator of political attitudes. Due to SDO, or political ideology as a broader term, being a potential confound with regards to ERP responses to political statements, the SDO of the current sample was compared with sample SDOs reported in other studies reporting culturally comparable samples (Table 1). See section 3.1 for results.

**Table 1:** Comparison of the SDO expression of the current study with previous studies.

Article	*M*	*SD*	*M_AGE_*	*SD_AGE_*	N	*t*	*df*	*p_adj_*
(Aiello et al., 2019)	2.41	1.04^a^	35.80	15.37	497	0.000	53.29	0.999
(Hons et al., 2026)	2.45	0.91	25.18	8.50	92	0.284	97.07	0.999
(Carvalho et al., 2025)	2.62	0.94	30.33	14.05	313	1.801	58.44	0.412
(Ho et al., 2015)—Sample 1	2.88	1.19	34.4	12.51	528	4.051	57.45	0.002
(Ho et al., 2015)—Sample 2	2.55	1.19	26.8	9.70	483	1.196	59.53	0.948
(Ho et al., 2015)—Sample 3	2.56	1.28	35.4	11.86	458	1.252	65.05	0.999
(Berry, 2023)^b^—SDO-D	2.66	1.24	—	—	57	0.262	86.16	0.999
(Berry, 2023)^b^—SDO-E	2.76	1.26	—	—	57	1.937	92.01	0.392
Current study—Global SDO	2.41	0.64	27.58	5.24	38	—	—	—
Current study—SDO-D	2.71	0.60	27.58	5.24	38	—	—	—
Current study—SDO-E	2.36	0.75	27.58	5.24	38	—	—	—

^a^SD was reported item-wise in the corresponding article. The lowest SD was used here.

^b^(Berry, 2023) represents a meta-analysis from which sample-size-weighted means and standard deviations were derived across *k* = 57 studies.

#### 2.2.4 Target words, statements and person descriptions

All target words, statements, and person descriptions were presented in German. The target words used in the study were derived from our previous investigation (Hons et al., 2026). In that online study (*n* = 92), two schematic avatars were presented, embodying left or right political orientations. For each word, participants were asked which political side, represented by the avatars, was more likely to use the word and to which extent. This was quantified by using a visual analogue scale (VAS) ranging from 0 (left-wing avatar) to 100 (right-wing avatar). Additionally, they were asked how derogatory they considered the word on a VAS from 0 (“not derogatory”) to 100 (“very derogatory”). From the 122 words evaluated in the previous study, 60 target words were selected for the current investigation, which were rated as particularly left or right (30 left, 30 right). Furthermore, target words were matched across coherence conditions with respect to word frequency, number of characters, number of syllables, and position in the statement. All used lexemes and corresponding lexical, morphological and evaluative values are provided in Supplementary Table S2. We derived the respective word frequencies from the “Gegenwartskorpora mit freiem Zugang” made accessible by the “Digitales Wörterbuch der Deutschen Sprache” (DWDS). This meta-corpus featured 4.8 million documents containing a total of 3.2 billion tokens, covering a time span from 1897 to 2026 at the time of the analysis. Four lexemes were not subjected to the matching procedure, as they had prominent homonyms that biased the frequency estimation: “Rechter” (C), “Integration” (C), “Linker” (IC), and “Grünling” (IC). The corresponding person descriptions were constructed in such a way that they cohered with left-rated lexemes and did not cohere with the right-rated lexemes. In our previous investigation (Hons et al., 2026) we found that right-wing lexemes were rated as more offensive than their left-wing counterparts, on average. The target words were embedded in statements, with the respective terms always presented in the center of the sentence. The statements were created by the study authors and addressed politically relevant topics such as environmental protection, migration, or social equality. The sentences contained first-person phrases such as “I think” or “in my opinion” in order to elicit greater emotional and cognitive involvement through personal reference. Before each statement, a brief person description was presented to formulate the context. The statement structure was as follows: A neutral beginning, a target word that should violate the given context, and the remaining sentence, holding the actual political statement, which was designed to be always coherent with the person description. Note that, for the analysis presented here, the actual political statements were irrelevant, as we were solely interested in extracting ERPs time-locked to the target word presentation. A total of 120 statements were presented: 60 statements with contextually coherent target words and 60 statements with incoherent target words. These consisted of two sets of the same 30 statements, once with the target word between quotation marks and once without quotation marks. In total, 13 person descriptions were created, which preceded the statements. In the experimental paradigm, gender-neutral German language was used for reasons of readability and to avoid potential biases in evaluations and neurophysiological response patterns due to changes in morphological complexity.

#### 2.2.5 Material not subjected to the analyses

Participants rated each statement in terms of their agreement, subjective sympathy of the speaker, and subjective competence of the speaker. These measures were excluded from the present analyses, as they were not relevant for addressing the research questions of this study. They were collected solely to enable potential future investigations that may build upon the findings reported in this paper.

### 2.3 Experimental procedure

**Figure 2:**
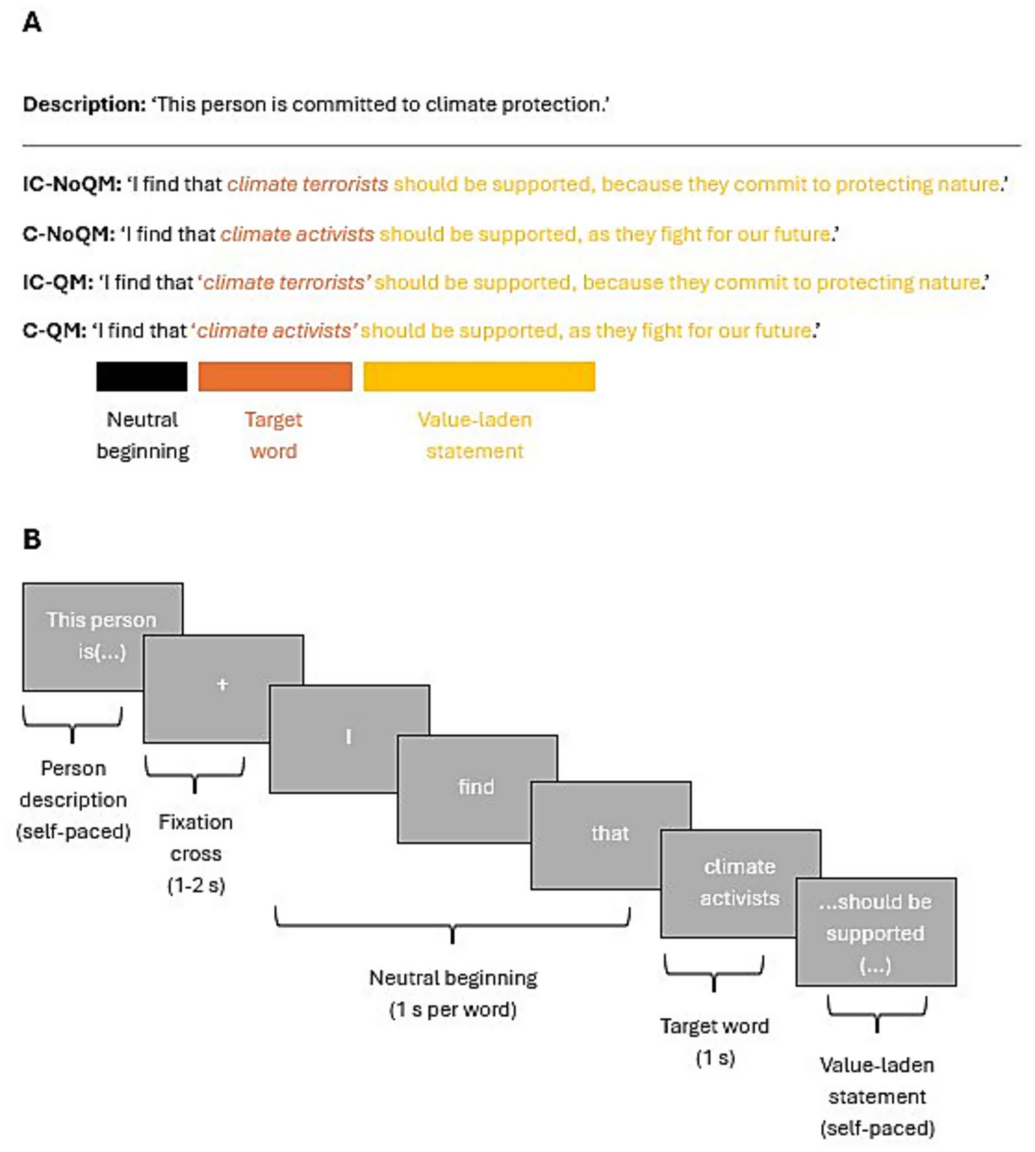
**(A)** Exemplary statements for all conditions. Note that statements were translated from German to English. **(B)** Schematic trial procedure. (.): placeholder added by the authors. IC-NoQM: Incoherent, without quotation marks. C-NoQM: Coherent, without quotation marks. IC-QM: Incoherent, with quotation marks. C-QM: Coherent, with quotation marks.

In the beginning of each session, sociodemographic questions and the SDO were administered. The core of the paradigm consisted of a fictitious person’s description, followed by a statement from that person (Figure 2). The statements varied in terms of two conditions: Coherence and Quotation. Coherence consisted of two levels characterized by either a contextually coherent or a contextually incoherent target word. In a second condition, Quotation, the target words are either enclosed in quotation marks or not. The sentences were presented on a computer screen, and the visual display was programmed using PsychoPy software (Peirce et al., 2019). Before the start of each statement, a fixation cross was displayed—the duration was randomly selected from the following list with uniform probability: [1, 1.2, 1.4, 1.6, 1.8, 2] (in s). Subsequently, the statements were presented in white text on a gray background. The first part of the statement, up to and including the target word, was presented word by word, with 1 s of duration per word. After the target word was displayed, the remaining part of the statement was shown all at once. The entire statement was presented for about 25 s. After each statement, participants were asked to rate how sympathetic they found the producer of the statement (S), how competent they perceived them (C), and to what extent they agreed with the statement (A)—all on a visual analog scale ranging from 0 (S: “not sympathetic at all,” C: “not competent at all,” A: “do not agree at all”) to 100 (S: “completely sympathetic,” C: “completely competent,” A: “completely agree”). These follow-up questions were self-paced and hence varied in duration. Note that, these self-report data were not subjected to the analyses performed here. After completing the EEG paradigm, the subjective Pejorative Weight of the target words was assessed by asking participants “How derogatory do you find the word?” on a visual analog scale from 0 (“not derogatory”) to 100 (“very derogatory”). Additionally, participants had to indicate whether they knew the meaning of the target words.

### 2.4 EEG preprocessing

The preprocessing of the recorded EEG raw data was performed using Brain Vision Analyzer 2.3 (Brain Products, Gilching) and Python 3.12.10. The former was used for the cleansing of the signals as well as segmentation and exportation of segments, while the latter was used to concatenate the data and extract the metrics used for N400 calculation. First, a new reference (linked mastoid reference) was created from the two reference electrodes. By means of visual inspection noisy electrodes were excluded from further processing. In the first processing step, the data were manually inspected for muscle artifacts, and these time segments were excluded. To filter out noise from the recordings, a notch filter at 50 Hz was applied. Additionally, a high-pass filter of 0.01 Hz was set to attenuate low frequencies, and a low-pass filter of 70 Hz was applied to suppress high frequencies. In the next step, ocular artifacts, such as movements, rolls, and blinks, were marked and removed using Independent Component Analysis. Subsequently, the data were semi-manually checked and corrected for remaining artifacts. The following criteria were considered: maximum allowable voltage difference between two data points = 50 μV/ms; length of intervals = 200 ms; maximum voltage difference within intervals = 200 μV; minimum amplitude = −150 μV, maximum amplitude = 150 μV; least activity (maximum—minimum) = 0.5 μV. For artifacts, the time interval from 200 ms before the artifact to 200 ms after the artifact was marked and subsequently manually removed. In the final step of preprocessing, the signal was segmented according to their stimuli or conditions. The segments began 200 ms before the presentation of the target word and ended 1,000 ms after the presentation of the target word.

### 2.5 ERP metric extraction

**Figure 3:**
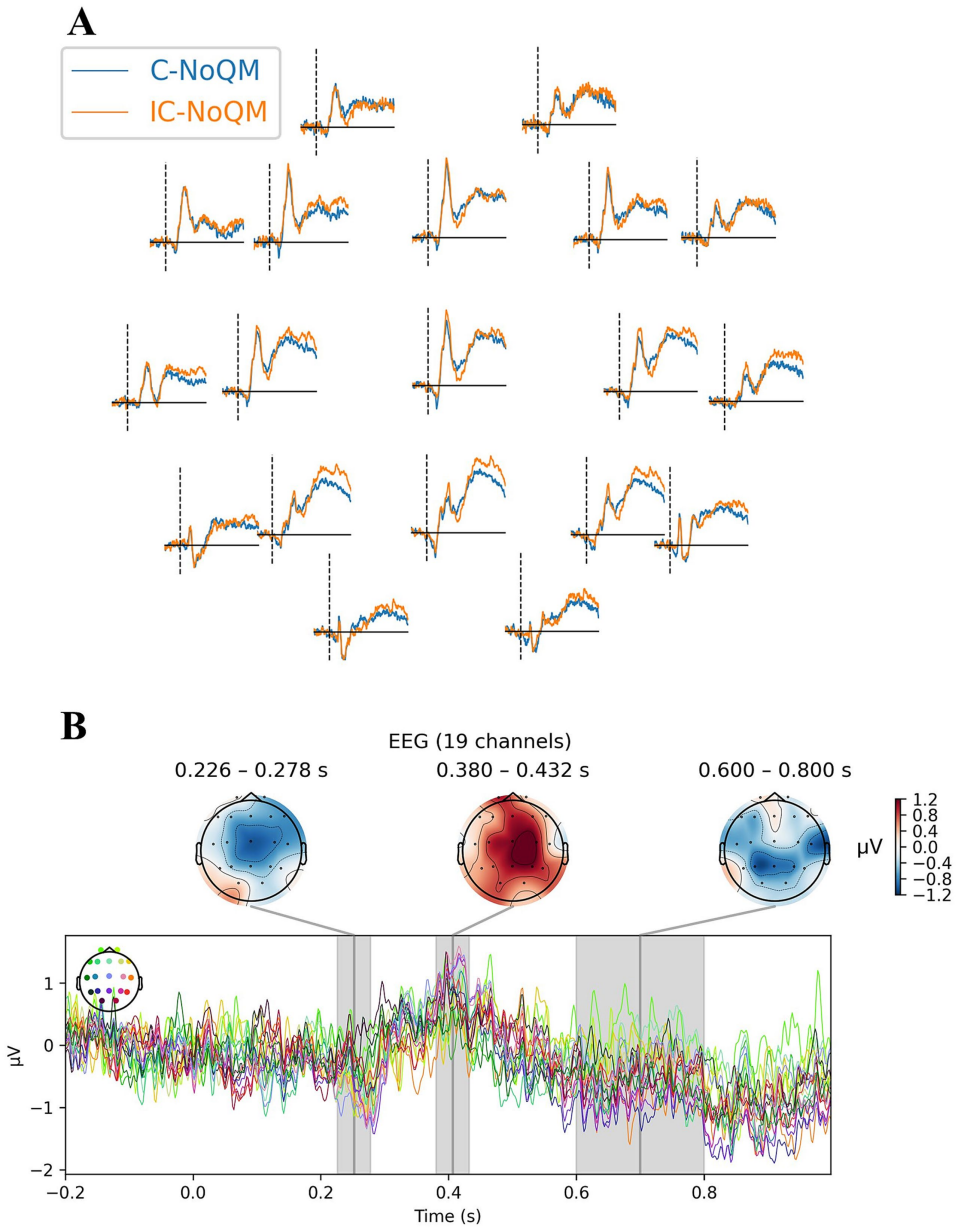
**(A)** Grand average time courses of the contextually coherent condition without quotation marks (C-NoQM) as well as the contextually incoherent condition without quotation marks (IC-NoQM) across all electrode positions. **(B)** Difference wave of C-NoQM and IC-NoQM grand averages and corresponding topoplots of averages of highlighted common ERP time windows.

**Figure 4:**
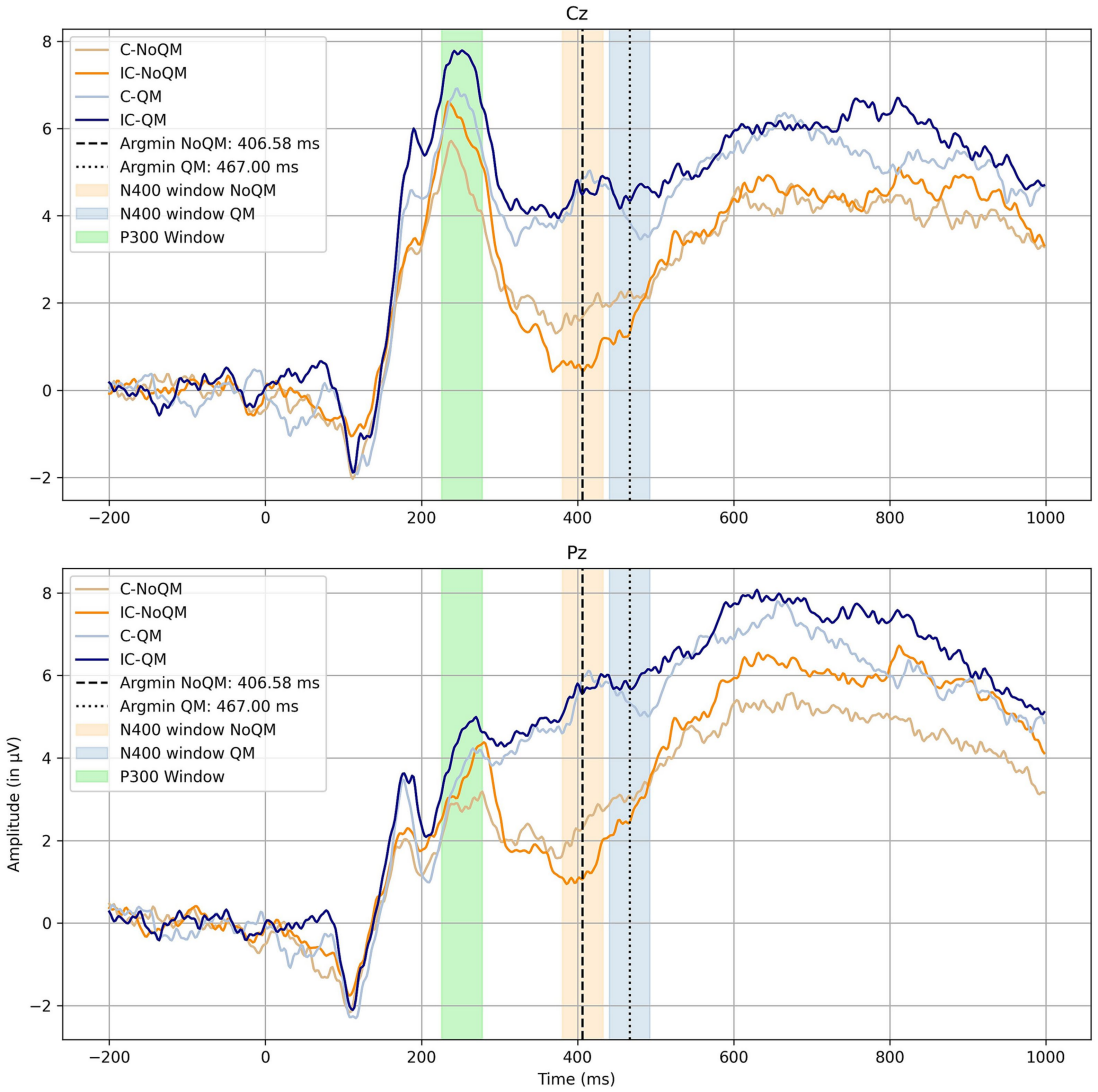
Grand average time courses for the two electrode positions of interest concerning the N400 and the P300 component across all conditions with the averages of the detected N400 arg mins and calculation windows of both components being highlighted.

Topographical maps were used to identify EEG electrodes exhibiting the expected ERP components (Figure 3). Grand-averaged waveforms for both conditions (Figure 3A), as well as their difference wave (Figure 3B), indicated that electrodes Cz and Pz were suitable for quantifying the N400 and P300 components. For the LPP, electrodes P3, Pz, and P4 appeared most appropriate. These observations are consistent with previous literature. Šoškić et al. (Šoškić et al., 2022) reported that Cz and Pz were among the most frequently selected electrodes for N400 quantification across 132 ERP studies. In addition, (Van Berkum et al., 2008), (Van Berkum, 2009) demonstrated that N400 amplitudes are typically maximal at centroparietal midline sites. Similarly, centro-parietal midline electrodes have been described as characteristic recording sites for both the P300 and the LPP components (Hajcak et al., 2010). Based on visual inspection of the grand-average ERP waveforms, a temporal shift of the N400 component was observed in the condition with quotation marks. To account for this, N400 time windows were determined individually for each Quotation condition and participant by means of a two-step process: I. The search for the peak and II. The extraction of the N400 by averaging a window spanning around said peak. Specifically, for each participant and each Quotation condition, ERPs were averaged across trials and electrodes, and the resulting signals were smoothed using a moving average filter. Then the latency of the minimum amplitude (arg min) within the pre-defined interval of 350–750 ms post-stimulus onset was identified. Note that, regarding the condition without quotation marks, the time courses representing incoherent statements were subjected to the peak search, while in the condition with quotation marks, the coherent pendants were used, as indicated by the presence of peaks in the area of interest (Figure 4). This procedure yielded a vector of 76 individual N400 arg mins (38 participants × 2 condition levels) yielding averages of 406.579 ms (NoQM) and 467.000 ms (QM). N400 values were then extracted by averaging a ± 26 ms window centered around the identified arg mins. Within each Quotation category, the identical N400 windows were applied to both Coherence categories. For the sake of completeness, we additionally computed an N400 model based on a fixed time-window for all conditions, ranging from 300 ms to 500 ms post-onset (Supplementary Table S3). The model showed a significant Coherence*Quotation interaction, but no significant differences between IC-NoQM and C-NoQM as well as IC-QM and C-QM (Supplementary Table S4). This was anticipated given the short peak windows and apparent time lag in the condition with quotation marks (Figure 4) and highlights the need for our data-driven peak search procedure.

For the P300 and LPP components, no temporal shifts were indicated in any condition. Therefore, P300 amplitudes were extracted by averaging a ± 26 ms window around the maximum amplitude (arg max) in the range of 200–400 ms of the grand-average ERP waveform, yielding center of the time window at 252 ms. LPP amplitudes were extracted by averaging across a pre-defined window between 600 and 800 ms.

### 2.6 Statistical analysis

All statistical analyses were conducted in R (version 4.5.0).

#### 2.6.1 Lexeme characteristics and SDO

To investigate whether coherence conditions differed in terms of meaningful lexeme characteristics, Welch’s t-tests were calculated for the following variables of interest: target word frequencies, number of characters of the target word, number of syllables of the target word, position of the target word in the sentence, and the pejorative weight of target words. Word frequencies were calculated as frequencies per million (FPM). *p*-values were corrected by means of Bonferroni-Holm adjustment. To determine whether SDO of this sample differed significantly from the reported sample SDO of other studies, the identical procedure was employed. As the number of syllables showed a trend towards significance (see 3.1), we separately performed a linear mixed effects model to test whether the number of syllables significantly predicted N400 amplitudes. As the grand average time courses indicated a flip in the N400 effect polarity, which could potentially mask a significant association between the number of syllables and N400 amplitudes, we ran the model solely on NoQM data. The model was built according to the maximal model that reached convergence in the main ERP analysis (see 2.6.2), which led to the following model structure: N400 amplitudes as the response variable, the number of syllables as the predictor, a by-subject random intercept and a slope parameter for the number of syllables, a by-item random intercept and random slope for the number of syllables, and a by-channel random intercept.

#### 2.6.2 ERP analysis

For our ERP analyses, we performed linear mixed effects models using the R package lme4 1.1 (Bates et al., 2015). Coherence (contextually coherent vs. incoherent) and Quotation (with vs. without quotation marks) functioned as our categorical fixed effect variables. Literature has reported substantial impact of insulting language (Struiksma et al., 2022) and emotional stimuli in general (Fields, 2023; Hajcak and Foti, 2020; Van Berkum et al., 2009) on the ERP response. Because a significant difference was found between coherent and incoherent target words in terms of pejorative weight, we decided to include the pejorative weight of the target words as a third and continuous fixed effect variable. All continuous variables, i.e., Pejorative Weight as well as the N400, P300 and LPP amplitudes, were z-standardized across all subjects and trials as this reduces multicollinearity and can help with convergence problems (Brauer and Curtin, 2018). In terms of the combinations of categorical effect levels, four distinct conditional categories can be derived: coherent/without quotation marks (C-NoQM), coherent/with quotation marks (C-QM), incoherent/without quotation marks (IC-NoQM), and incoherent/with quotation marks (IC-QM). We will use this notation throughout the manuscript. Adhering to the agenda proposed by Barr and colleagues (Barr et al., 2013) as well as (Brauer and Curtin, 2018), we aimed at the maximal random effects structure implied by our complex design: by-subject random intercepts, and by-subject random slopes for main effects of Coherence, Quotation and Pejorative Weight as well as their interactions, since all are within-subject predictors. Furthermore, by-item random intercepts, and by-item random slopes for Quotation and Pejorative Weight. Coherence did not contribute a random slope here, as the items do not repeat across Coherence categories, i.e., each category has its own specific set of items, which means both are independent on an item basis. Note that items refer to the target words here. Finally, by-channel random intercepts as well as by-channel random slopes for Coherence, Quotation and Pejorative Weight as well as their interaction were specified. The maximum iteration parameter was set to 100,000 and the optimizer of choice was “bobyqa.” For post-hoc testing we used emmeans 1.11. with the Bonferroni-Holm method as the means for alpha error correction. If the maximal model would not converge, we would assess the model output, such as correlation tables, to derive the most effective steps to further simplify the model structure. All three maximal ERP models showed multicollinearity of channel-wise random effects variables, which is why we simplified the channel-wise random effects term by removing the slopes. If the model would still not converge, we would modify the interactions of the random effect terms to remove the slopes of the predictors and their lower-level interactions, while keeping the slope of the higher-order interaction (Barr et al., 2013). With this system of simplification, the N400 model and the LPP model reached convergence. Regarding the P300 model, by-subject collinearity in the random slopes across quotation conditions was detected. To mitigate this issue, we removed the random slopes of quotation from the within-subjects random effect term. Using this simplified specification, the P300 model converged, too. Normality of residuals, normality of random effects and homoskedasticity were supported by the data. Proof is provided via the publicly available R-code in our repository. Finally, to account for the potential confounding influence of Social Dominance Orientation on the N400, the corresponding linear mixed effects model was replicated and modified: The simple participant coding was replaced by the individual average SDO-7 scores (Supplementary Table S5). Both models produced the same significant effects, indicating no reason to assume confounding effects imposed by Social Dominance Orientation. Since the model with the simple participant coding provided better model explanation as quantified by the Akaike Information Criterion, we used this model to report results from.

## 3 Results

The following section first reports the characteristics of the lexemes and samples, followed by a detailed presentation of the ERP model outcomes. *p*-values ≤ 0.06 that did not reach conventional significance thresholds were interpreted as indicating a trend toward significance and are therefore described in greater detail than clearly non-significant findings. In addition, a more extensive report and follow-up analysis were conducted for the Number of Syllables variable, as its associated *p*-value (0.061) approached significance and warranted further examination to exclude a potential influence on N400 amplitudes.

### 3.1 Lexeme characteristics and SDO

No significant differences between incoherent and coherent target words were present in terms of Word Frequency (*t*_37.17_ = 0.557, *p* = 0.581), Number of Characters (*t*_57.62_ = 1.482, *p* = 0.432), Number of Syllables (*t*_53.95_ = 2.508, *p* = 0.061), and Position in the Statement (*t*_49.51_ = 1.143, *p* = 0.517). Only in terms of Pejorative Weight a significant difference was found: Incoherent target lexemes (*M* = 32.484, *SD* = 23.312) were rated as more derogatory than their coherent counterparts (*M* = 61.354, *SD* = 23.003, *t_57.99_* = 4.828, *p* < 0.001). For all means and standard deviations of the lexeme characteristics of interest, refer to Table 2. As the Number of Syllables indicated a trend towards significance, we tested whether a significant relationship with the N400 response could be found: A linear mixed effects model assessing the predictive power of the number of syllables on the N400 amplitude indicated no significant effect (*b* = 0.037, *SE* = 0.024, *t_67.61_* = 1.571 *p* = 0.121).

**Table 2:** Descriptive statistics of both coherence conditions for the lexeme-related variables of interest.

Condition	*M* (*SD*)				
	Frequency	Number of characters	Number of syllables	Position of target	Pejorative Weight
Coherent	3.68 (9.42)	11.60 (3.99)	4.23 (1.57)	4.73 (0.69)	32.48 (23.31)
Incoherent	6.13 (21.31)	10.13 (3.67)	3.33 (1.18)	4.47 (1.07)	61.35 (23.00)

Regarding SDO, the current sample differed significantly from only one out of eight other studies with culturally comparable samples (Table 1): Ho et al. (2015) reported significantly higher SDO-7 scores from a sample native to the United States (*M* = 2.88, *SD* = 1.19) compared to the sample of the current study (*M* = 2.41, *SD* = 0.64, *t_57.45_* = 4.051, *p* = 0.002). The remaining contrasts were insignificant. Comparatively, the SDO scores reported by Ho et al. (2015) differed significantly from six out of eight possible contrasts.

### 3.2 ERP model results

Amplitudes are reported as “more pronounced” when they reflect the canonical direction of the respective component, i.e., more negative deflections are considered more pronounced in the N400 domain, whereas more positive deflections are considered more pronounced in the P300 and LPP domain. For raw ERP means and standard deviations, refer to Table 3.

**Table 3:** Channel-wise amplitude means (in μV) and standard deviations for each condition and component.

Component	Coherence	Quotation	Channel	Mean amplitude	SD amplitude
N400	C	NoQM	Cz	1.439	11.475
			Pz	2.013	11.469
		QM	Cz	1.992	12.798
			Pz	3.095	12.783
	IC	NoQM	Cz	−0.633	11.899
			Pz	0.081	11.566
		QM	Cz	3.243	12.387
			Pz	4.469	12.758
P300	C	NoQM	Cz	4.986	10.189
			Pz	2.724	9.622
		QM	Cz	6.479	11.150
			Pz	3.513	10.842
	IC	NoQM	Cz	5.839	10.676
			Pz	3.308	10.547
		QM	Cz	7.571	10.937
			Pz	4.477	11.019
LPP	C	NoQM	P3	4.416	11.811
			P4	4.572	13.170
			Pz	5.009	13.156
		QM	P3	5.528	12.687
			P4	5.551	13.585
			Pz	6.961	13.611
	IC	NoQM	P3	5.461	12.193
			P4	5.449	12.954
			Pz	6.054	13.036
		QM	P3	6.639	12.402
			P4	6.524	13.394
			Pz	7.623	13.626

#### 3.2.1 N400 temporal analysis

With a mean delay of 60.421 ms the N400 latency of the condition with quotation marks (*M* = 467.000, *SE* = 19.910) showed substantial time lag compared to the condition without quotation marks (*M* = 406.579, *SE* = 8.240, *t_37_* = −3.54, *p* = 0.001, see Figure 4).

#### 3.2.2 N400 amplitude analysis

**Figure 5:**
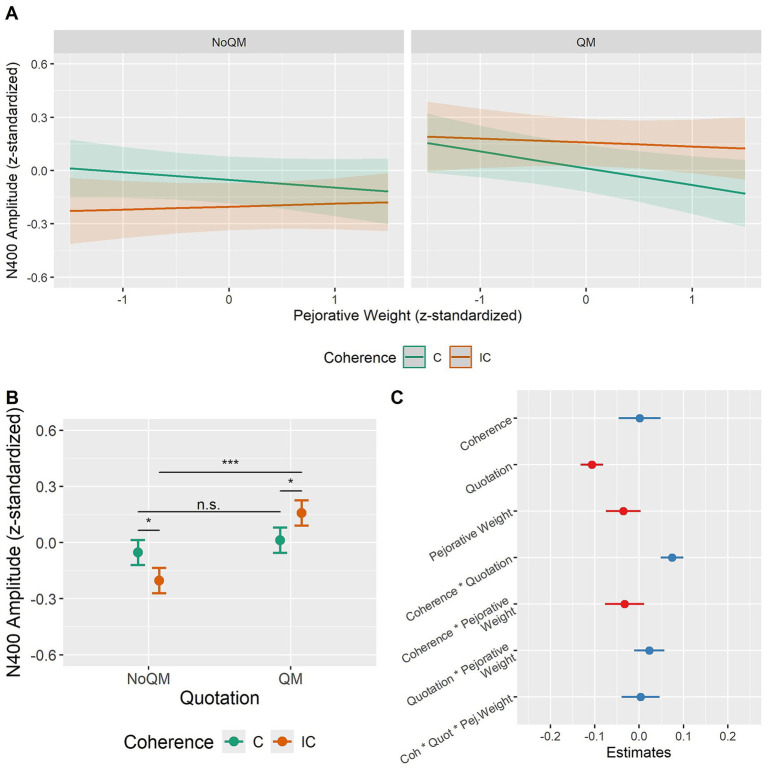
N400 amplitude results visualization. **(A)** *Pejorative Weight* slopes across all combinations of *Coherence* and *Quotation.* **(B)** N400 amplitude means (in μV) across the conditions *Coherence* and *Quotation*. Error bars indicate standard errors. **(C)** Fixed effect estimates of all main effects and interactions. Error bars indicate 95% confidence intervals. *** < 0.001, ** < 0.01, * < 0.05, n.s. > 0.05.

The predictor Quotation (*b* = −0.107, *SE* = 0.013, *t_775.83_* = −8.292, *p* < 0.001, *η^2^_p_* = 0.08) reached significance. The effects of Coherence (*b* = 0.001, *SE* = 0.024, *t_61.73_* = 0.055, *p* = 0.741, *η^2^_p_* < 0.01) and Pejorative Weight (*b* = −0.036, *SE* = 0.020, *t*_61.33_ = −1.805, *p* = 0.076, *η^2^_p_* = 0.05) on N400 amplitude did not indicate significance. Since the predictor Quotation was involved in a significant categorical interaction, interpretation of the effects is provided below. The interaction term Coherence*Quotation (*b* = 0.074, *SE* = 0.013, *t_775.52_* = 5.746, *p* < 0.001, *η^2^_p_* = 0.04) reached significance (Figure 5B): Post-hoc tests indicated that contextually incoherent target words (*M* = −0.203, *SE* = 0.067) produced more pronounced, i.e., numerically lower, N400 amplitudes than contextually coherent target words (*M* = −0.052, *SE* = 0.067, *z.ratio_inf_* = 3.022, *p* = 0.016) when no quotation marks were present. With quotation marks the pattern flipped: contextually coherent target words (*M* = 0.013, *SE* = 0.067) yielded more pronounced N400 amplitudes compared to their contextually incoherent pendants (*M* = 0.158, *SE* = 0.067, *z.ratio_inf_* = −2.706, *p* = 0.016). Further, while the difference between incoherent targets with and without quotation marks turned significant (*z.ratio_inf_* = −9.978, *p* < 0.001), the same contrast did not yield significance for coherent targets (*z.ratio_inf_* = −1.598, *p* = 0.073). The remaining interactions Coherence*Pejorative Weight (*b* = −0.033, *SE* = 0.022, *t_59.62_* = −1.467, *p* = 0.148, *η^2^_p_* = 0.03), Quotation*Pejorative Weight (*b* = 0.023, *SE* = 0.017, *t_51.92_* = 1.302, *p* = 0.199, *η^2^_p_* = 0.03) as well as the three-way interaction Coherence*Quotation*Pejorative Weight (*b* = 0.003, *SE* = 0.022, *t_50.47_* = 0.151, *p* = 0.881, *η^2^_p_* < 0.01) did not produce significant results (Figure 5A). All fixed effect estimates are visually summarized in Figure 5C.

#### 3.2.3 P300 amplitude analysis

**Figure 6:**
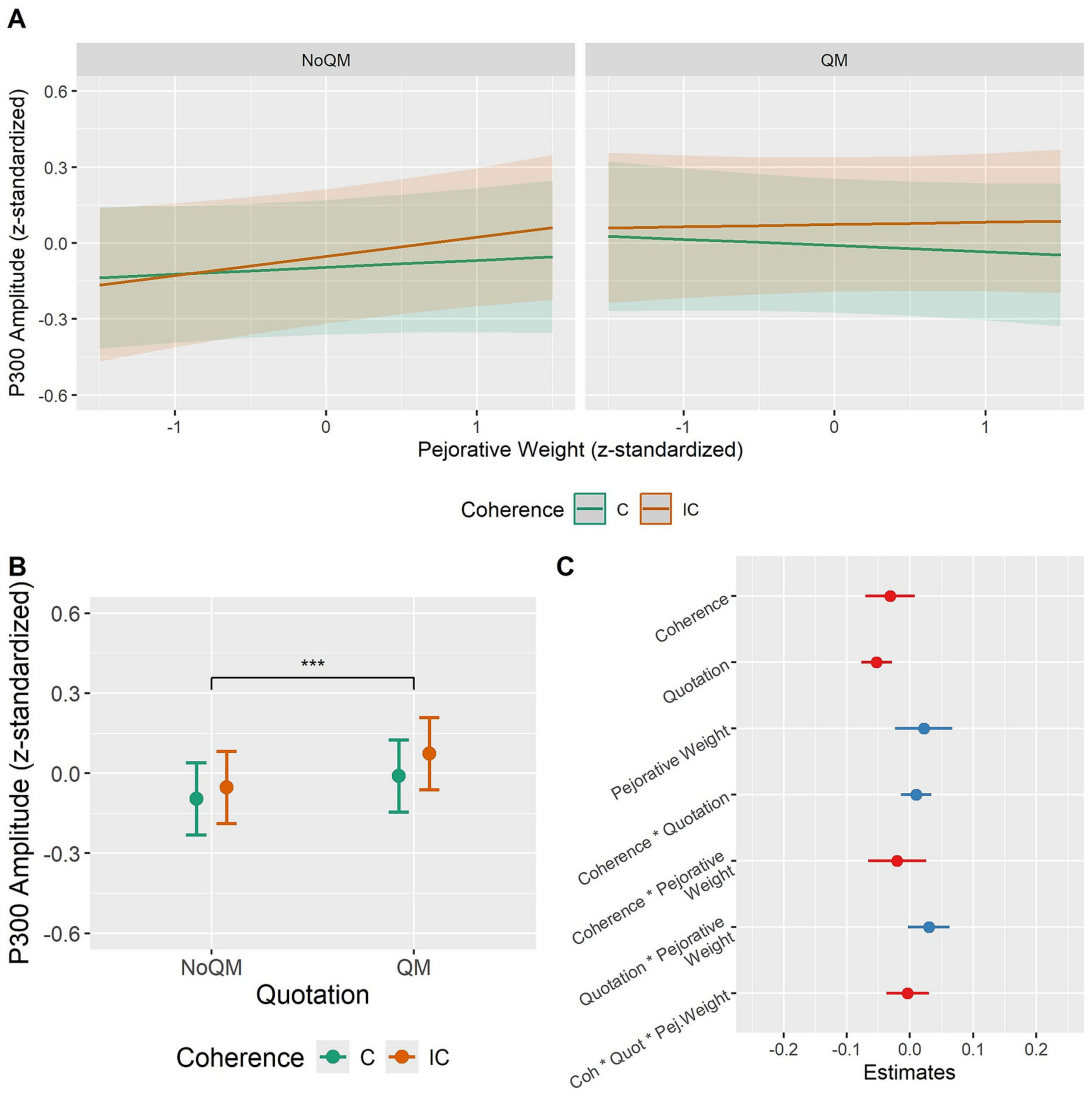
P300 amplitude results visualization. **(A)** *Pejorative Weight* slopes across all combinations of *Coherence* and *Quotation.* **(B)** P300 amplitude means (in μV) across the conditions *Coherence* and *Quotation*. Error bars indicate standard errors. **(C)** Fixed effect estimates of all main effects and interactions. Error bars indicate 95% confidence intervals. *** < 0.001, ** < 0.01, * < 0.05, n.s. > 0.05.

Quotation showed a significant effect (*b* = −0.053, *SE* = 0.012, *t_804.44_* = −4.302, *p* < 0.001, *η^2^_p_* = 0.02; Figure 6B) on P300 amplitude: Target words with quotation marks (*M* = 0.032, *SE* = 0.133) exhibited significantly more pronounced P300 amplitudes than target words without quotation marks (*M* = −0.074, *SE* = 0.133). Coherence (*b* = −0.032, *SE* = 0.020, *t_61.10_* = −1.587, *p* = 0.118, *η^2^_p_* = 0.04), Pejorative Weight (*b* = 0.022, *SE* = 0.023, *t_56.99_* = 0.954, *p* = 0.344, *η^2^_p_* = 0.02) as well as the interactions Coherence*Quotation (*b* = 0.010, *SE* = 0.012, *t_796.51_* = 0.806, *p* = 0.420, *η^2^_p_* < 0.01; Figure 6B), Coherence*Pejorative Weight (*b* = −0.020, *SE* = 0.023, *t_52.69_* = −0.875, *p* = 0.386, *η^2^_p_* = 0.01), Quotation*Pejorative Weight *(b* = 0.030, *SE* = 0.017, *t_56.27_* = 1.777, *p* = 0.081, *η^2^_p_* = 0.05; Figure 6A) and Coherence*Quotation*Pejorative Weight (*b* = 0.006, *SE* = 0.018, *t_47.54_* = 0.333, *p* = 0.741, *η^2^_p_* < 0.01; Figure 6A) failed to reach significance. All fixed effect estimates are visually summarized in Figure 6C.

#### 3.2.4 LPP amplitude analysis

**Figure 7:**
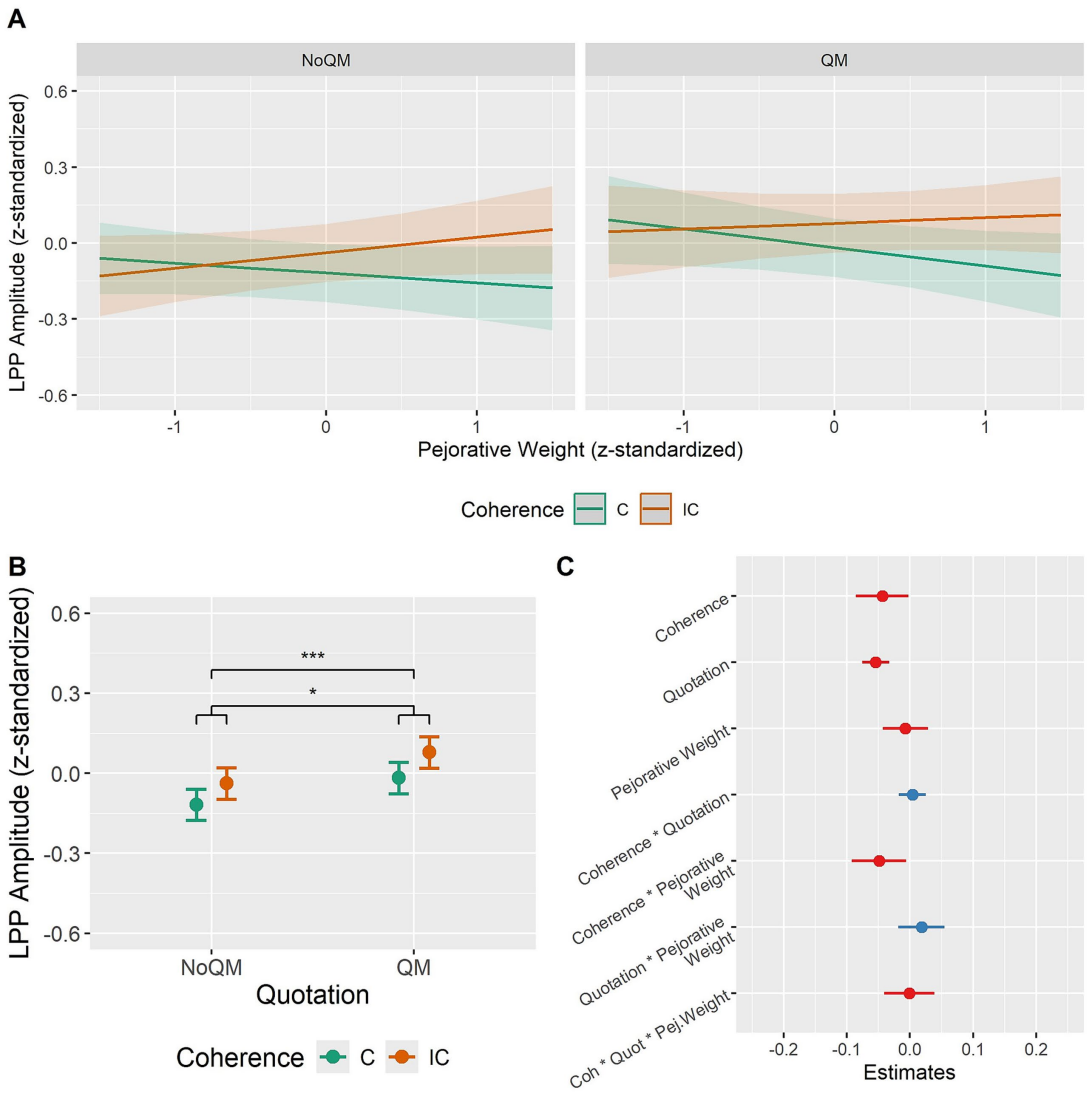
LPP amplitude results visualization. **(A)** *Pejorative Weight* slopes across all combinations of *Coherence* and *Quotation.* **(B)** LPP amplitude means (in μV) across the conditions *Coherence* and *Quotation*. Error bars indicate standard errors. **(C)** Fixed effect estimates of all main effects and interactions. Error bars indicate 95% confidence intervals. ***p < 0.001, **p < 0.01, *p < 0.05, n.s. > 0.05.

The main effect Quotation proved significant (*b* = −0.054, *SE* = 0.011, *t_1011.31_* = −4.990, *p* < 0.001, *η^2^_p_* = 0.02; Figure 7B), with target words enclosed in quotation marks (*M* = 0.030, *SE* = 0.054) exhibiting higher LPP amplitudes than target words without quotation marks (*M* = −0.078, *SE* = 0.054). Further, the predictor Coherence showed a significant effect (*b* = −0.044, *SE* = 0.021, *t_62.09_* = −2.081, *p* = 0.042, *η^2^_p_* = 0.07; Figure 7B), with incoherent target words (*M* = 0.020, *SE* = 0.057) showing more pronounced LPP amplitudes than their coherent counterparts (*M* = −0.068, *SE* = 0.057). The predictor Pejorative Weight remained insignificant (*b* = −0.007, *SE* = 0.018, *t_62.65_* = −0.398, *p* = 0.692, *η^2^_p_* < 0.01). The interaction term Coherence*Pejorative Weight turned significant (*b* = −0.049, *SE* = 0.022, *t_61.49_* = −2.225, *p* = 0.030, *η^2^_p_* = 0.07): Contextually incoherent target words (*M* = 0.042, *SE* = 0.030) showed a more positive Pejorative Weight trend than contextually coherent words (*M* = −0.056, *SE* = 0.027; Figure 7A). The interaction terms Coherence*Quotation (*b* = 0.004, *SE* = 0.011, *t_1006.49_* = 0.371 *p* = 0.711, *η^2^_p_* < 0.01; Figure 7B), Quotation*Pejorative Weight (*b* = 0.018, *SE* = 0.019, *t_49.49_* = 0.979, *p* = 0.332, *η^2^_p_* = 0.02) and Coherence*Quotation*Pejorative Weight (*b* = −0.001, *SE* = 0.020, *t_51.67_* = −0.059, *p* = 0.954, *η^2^_p_* < 0.01; Figure 7A) failed to reach significance. All fixed effect estimates are visually summarized in Figure 7C.

## 4 Discussion

The primary aim of this study was to provide evidence for the existence of the extensively researched N400 effect in a socio-pragmatic political discourse setting. Participants were confronted with descriptions of fictitious people and corresponding political statements containing target words that were either coherent or incoherent with the previously provided person description. We anticipated the emergence of an N400 effect, which would be characterized by a larger negative deflection at ~400 ms after the onset of contextually incoherent target words compared to contextually coherent ones. Furthermore, we explored the potential cancelling of this effect by deploying quotation marks to the target words of the political statements.

In line with previous findings (Filik and Leuthold, 2008; Kulakova and Nieuwland, 2016; Van Berkum et al., 1999; Van Berkum et al., 2009), the anticipated standard N400 effect could be observed in the current study: Pragmatic violations, as induced by a cognitive mismatch between a person descriptions (e.g., “This person is committed to climate protection.”) and a target word in a political statement (e.g., “I find that *climate terrorists* should be supported, because they commit to protecting nature.”), were able to elicit a larger negative deflection after 400 ms on average compared to contextually coherent statements (e.g., “I find that *climate activists* should be supported, as they fight for our future.”). The existence of an N400 effect has been demonstrated in various domains: textual stimuli both on a sentence and a wider discourse level (Van Berkum et al., 1999; Van Berkum et al., 2003), mental calculation problems (Niedeggen et al., 1999), brand logos (Dini et al., 2022) as well as musical sequences (Calma-Roddin and Drury, 2020) were found to elicit an N400 response pattern. Prior studies have shown that implicit socio-pragmatic speaker characteristics (e.g., gender, age) can create a contextual framework, and violations of this context can elicit an N400 effect (Hanulíková and Carreiras, 2015; Van Berkum et al., 2009; Van Berkum et al., 2008; Wu and Cai, 2025). This is comparable to the current study, where the contextual violation arises from a mismatch between the inferred social identity and the usage of a conflicting target word, rather than explicit pragmatic contradictions. The contextual violation therefore only occurs due to predefined cognitive models and stereotypes: Participants infer the social identity of a person and derive expectations in terms of language style based on their cognitive model of the respective social group. In summary, the N400 effect can be understood as the increased difficulty of integrating the current word into the pragmatic context (Jiang et al., 2013a).

In an exploratory attempt to cancel the evoked N400 effect, we additionally presented the statements with target words enclosed between quotation marks. This produces a reversal in the polarity of the N400 effect, as it led to a larger negative deflection when target words were contextually coherent compared to incoherent counterparts. We explain this result as follows: Quotation marks are understood as pragmatic markers, which carry the ability to alter the meaning of language. These metacommunicative markers can signal sensibility to, or distancing from, the content of lexemes by signaling that the producer of a word does not agree with its original meaning (Dreesen, 2019; Predelli, 2003). In everyday language, these are referred to as “scare quotes” (Predelli, 2003). When using quotation marks in conjunction with contextually incongruent lexemes, facilitation of pragmatic integration might occur by indicating a more context-aligning alternative meaning. To follow up on the previous example, “*climate terrorists*” might signal the distancing from the original meaning and imply an alternative meaning, namely that *climate terrorists* are not actually terrorists. This interpretation would be more consistent with the description of a speaker being a proponent of climate protection (“This person is committed to climate protection.”). Using contextually congruent words, on the other hand, might cause difficulties in pragmatic integration by implying an alternative meaning to an otherwise contextually valid target word. The expression *“climate activists”…* for instance, can be understood to mean that the producer rejects the stereotypical interpretation and thereby implying that climate activists are not activists at all, i.e., do not actively engage for a higher political good. This result would then be understood as a contextual violation, as the updated notion of the congruent lexeme conflicts with the speaker description of a climate protection proponent, which would then results in a flip in the N400 effect polarity. Together with the significant time delay observed during the processing of target words enclosed in quotation marks, our summarized N400 results suggest the following mechanism: Target words without quotation marks produce the conventional N400 pattern of more pronounced amplitudes in response to contextually incoherent versus coherent ones. When target words appear enclosed in quotation marks, our evidence suggests that regular semantic processing is suppressed and delayed. We argue that the additional step of updating the meaning of a word is responsible for the time delay in the neural occurrence of the N400 effect. Supportive of this finding, previous investigations demonstrated that additional cognitive processing costs during a pragmatic language task led to a temporal shift of the N400 (Hohlfeld et al., 2004b). Prior findings indicate that the interruption of the interpretation of speech by additional tasks leads to attentional shifts causing disturbances in the processing of a syntactic structure (Caplan and Waters, 1999), which are also understood to occur on a more fine-grained semantic level: In line with the works of Hohlfeld et al. (2004b), our results indicate that these disruptions already occur at the semantic word processing stage. By quoting a target word, the semantic processing of a word gets interrupted as meaning needs to be adjusted, before eventually propagating to the contextual integration of the “new” interpretation. We argue that the updated semantic meaning of a target word potentially facilitated the contextual integration of incoherent information, while making the integration of contextually congruent information more difficult. This mechanism may, in turn, account for the reversal in N400 effect polarity observed in response to target words presented in quotation marks. One might argue, however, that this effect could have also been driven by other factors, such as an increase in visual/morphological complexity or attentional shifts. If the effect were driven by increased visual complexity, one would expect comparable processing changes for both coherent and incoherent words, which would not account for a reversal in effect polarity. Likewise, a non-specific shift in attentional resources or metalinguistic processing would be expected to influence both word categories similarly, and there is currently no empirical evidence suggesting potential differential effects depending on pragmatic coherence. Finally, the observed differential modulation of the N400—extending to a reversal of the effect direction—suggests an interaction between quotation marks and the semantic properties of the target word. We therefore propose that quotation marks modulate N400 amplitudes as a function of word coherence. Overall, we consider this interpretation to be the most consistent with the present data.

Literature describes both P300 and LPP as responsive to significant stimuli, where significance can be substantiated by a variety of factors, such as emotional salience or stimulus infrequency (Brown et al., 2012; Fields, 2023; Hajcak and Foti, 2020; Huang and Luo, 2006). In the current study, we therefore hypothesized that Pejorative Weight predicts P300 as well as LPP amplitudes. Overall, this hypothesis could not be confirmed. While the general consensus is that the P300 and LPP components are generally modulated by emotionally salient stimuli (Hajcak and Olvet, 2008; Song et al., 2022), there are reports that could not confirm these links (Carretié et al., 1997; Yang et al., 2025). Yang et al. (2025), for instance, did not find the hypothesized LPP effect in an implicitly emotional task. The authors argued that due to the implicit emotional nature of the task, the elicitation of the LPP component might have failed. This may also explain the lack of LPP/P300 presence in the current study: During the EEG paradigm, our participants were instructed to assess whether they would agree with a given political statement and how sympathetic/competent they find the producer, rather than evaluate the emotional salience of the statements. The evaluation of the Pejorative Weight took place thereafter and was disconnected from the actual statement presentation and EEG paradigm. Given the lack of explicit instructions to emotionally evaluate the stimuli in the current study, this may explain why the hypothesized ERP components did not materialize. Furthermore, our analysis on potential P300 and LPP effects showed the following results: Quotation was found to have a significant effect on both components, with target words enclosed in quotation marks eliciting higher average amplitudes in both models. This might simply be explained by increased morphological complexity and resulting processing costs of target words with quotation marks (Hauk and Pulvermüller, 2004). The predictor Coherence also showed a significant effect on LPP amplitudes: Contextually incoherent target words showed increased responses compared to their coherent pendants. This is consistent with earlier research: Van Berkum (2009), for example, reported that value-inconsistent political statements produced not only an N400 effect, but also showed higher late positivity (500–650 ms) amplitudes. The authors interpret their findings as evidence of a heightened affective response to value-inconsistent stimuli. Translating to the current study, participants may have experienced a conflict between contextually incoherent target words and their own moral value system irrespective of the Pejorative Weight of the words. With regards to the functional connectivity of both components, there is ample evidence for the joint elicitation of the P300 and the LPP in response to emotional stimuli (Fields, 2023; Struiksma et al., 2022; Van Berkum et al., 2009). In the current study, we were only partially able to replicate these findings: The only shared result pattern observed was that Quotation showed significant predictive power on both components. With regards to a functional background of the early and late positivity, some authors argue that both stem from the same neurophysiological processes (Fields, 2023; Hajcak and Foti, 2020) or that they represent two temporal epochs of the very same ERP component (Van Berkum et al., 2009), since both components were found to behave so similarly. However, we also found evidence speaking against a shared functional background: The LPP model showed both a significant Coherence predictor and a significant Coherence*Pejorative Weight interaction, while not being present in the P300 model. This contrast suggests a more diffuse response pattern of the LPP compared to the P300. As of now, we do not have a well-founded explanation for these findings. More research will be needed to soundly investigate the concrete shared and non-shared mechanisms behind P300s and LPPs in response to emotionally salient, value-inconsistent textual stimuli.

In terms of limitations of the current study, contextually coherent and incoherent target words differed significantly in terms of their perceived Pejorative Weight. Although we addressed this fact by incorporating Pejorative Weight as a fixed effect predictor into the analysis, one cannot rule out any residual confounds. As Pejorative Weight was quantified using self-assessment measures, it is possible that it did not fully capture the variance attributed to the neural response to target words varying in their offensiveness. Similarly, political ideology may be a potential confounding factor. Despite having computed an additional model with the SDO scores of participants as random factor, one cannot fully rule out potential influences of political attitudes. As SDO represents no direct measure of political attitude but a self-report questionnaire that is correlated with a range of political positions (see section 2.2.3.) it is possible that the full impact of the political ideology of the participants has not been captured. Secondly, the topics and structures of the political statements varied across Coherence conditions. Therefore, neural variation attributable topic-specific differences cannot be ruled out. Further, the chosen lexeme presentation interval of 1,000 ms is longer than that of comparable studies. The rationale behind this decision was to accurately extract LPPs and avoid smearing with early ERP components of successive trials. And yet, this leaves more time for in-depth analysis of word meanings, potentially confounding the Quotation effect found in the current study. Furthermore, we would like to mention the circularity in our data-driven peak detection method: Since we did not anticipate the reversal and lag of the N400 effect when quotation marks were used, we performed the detection based on the conditions that showed a pronounced N400 peak in the grand average time courses (IC-NoQM & C-QM). As our analysis was based on a uniform time window for all conditions indicated, this was necessary to accurately capture the interactions witnessed in the grand average time courses. On the downside, this introduced data-driven circularity, as the conditions to perform the peak search on were those that showed a visible peak in the first place. Moreover, in terms presentation modality, we would like to mention that the usage of merely fictional characters producing the utterances under investigation might limit the generalizability of the results. Finally, to promote model convergence we subjected only a limited number of electrodes to the analyses, which was Cz and Pz in the N400 and P300 analyses, and P3, Pz and P4 in the LPP analysis. Although it is well established that more complex random-effects structures in linear mixed-effects models are associated with an increased risk of convergence problems (Liu et al., 2024), a comprehensive characterization of ERP components requires consideration of their full scalp distribution. In the present study, this was addressed only at a descriptive level (Figure 3).

In summary, contextual violations, here characterized as mismatches between descriptions of fictional characters and the target words they produced within political statements, yielded a lower amplitude after 400 ms than contextually coherent political statements. When target words were put between quotation marks the pattern flipped: In opposition to the classical N400 effect, contextually coherent target words enclosed in quotation marks elicited a lower N400 amplitude than contextually violating target words. Additionally, quotation marks caused a delay in the N400 time course that can be explained by the additional processing step in the form of updating the original meaning of a target word. In both the LPP and the P300 model, a significant effect of Quotation was found, with words enclosed in quotation marks separately showing higher amplitudes than their counterparts.

## Data Availability

The datasets presented in this study can be found in online repositories. The names of the repository/repositories and accession number(s) can be found in the article/Supplementary material.
