# Supplementary material for: The neural dynamics of political socio-pragmatic violations: an ERP study
Source: Front Hum Neurosci. 2026 Jun 29;20:1820376. doi: 10.3389/fnhum.2026.1820376 (PMC13357823; doi:10.3389/fnhum.2026.1820376)
Supplement: Supplementary file 1 [file Table_1.docx]

**Supplementary Table S1.** Deviations from the pre-registration.

| # | Details | | | Original Wording | Deviation Description | Reader Impact |
| --- | --- | --- | --- | --- | --- | --- |
| 1 | Type | Reason | Timing | “ERP amplitudes - especially in the range of ~250 ms to ~550 ms - time-locked to the presentation of the critical words” | Since a time shift was observed in the condition with quotation marks, we re-defined the N400-window of interest as 350-750 ms post stimulus onset. Further, the time windows for the P300 and LPP were set between 200-400 ms and 600-800 ms, respectively. | Altering the analysis windows of ERP components can have large implications on their calculation. However, ERPs time courses are difficult to predict precisely. In this instance, we did not expect to observe a time shift in the N400 response when a target word was put between quotation marks, as the research we used as a basis for this study did not indicate that this mechanism would occur.  Secondly, we additionally explored whether differences between conditions existed beyond the N400 component. We performed investigations in the P300 and LPP domains, as visual inspection suggested potentially interesting effects in these areas. |
|  | Analysis | New knowledge | After data access |  |  |  |
| 2 | Type | Reason | Timing | I: “In a second leg of analysis, we will investigate whether the agreement to political statements can be predicted by the sympathy and the perceived competence of a speaker, as well as the derogatory intensity and the probability of using a critical word.”  II: “Linear mixed effects model with the self-rated agreement values as criterion and the self-reported sympathy and competence values with respect to the speaker, as well as the self-reported derogatory intensities of the critical words and the probabilities of using them in everyday discourse as fixed factors, and the critical words as random effects.” | As we have observed that both domains, ERP and self-reports, are rather comprehensive on their own, we decided to produce two separate manuscripts. | The manuscript does not include any analyses concerned with self-report measures. Although this represents a substantial deviation to the pre-registration, readers can expect a comprehensive and self-contained work. |
|  | Variables | Other (see ‘Deviation Description’) | After data access |  |  |  |
