# Supplementary material for: The neural dynamics of political socio-pragmatic violations: an ERP study
Source: Front Hum Neurosci. 2026 Jun 29;20:1820376. doi: 10.3389/fnhum.2026.1820376 (PMC13357823; doi:10.3389/fnhum.2026.1820376)
Supplement: Supplementary file 2 [file Table_2.docx]

**Supplementary Table S2.** Target words and corresponding word characteristics.

| Target word | Coherence | Absolute frequency | Word frequency per million | Number of letters | Number of syllables | Target Position | Pejorative Weight | Pejorative Weight (survey)^a^ | Political connotation (survey)^a^ |
| --- | --- | --- | --- | --- | --- | --- | --- | --- | --- |
| Misogyn | C | 12 | 0.004 | 9 | 4 | 3 | 29.16 | 49.43 | 22.79 |
| Vielfalt | C | 68533 | 21.519 | 8 | 2 | 5 | 6.18 | 8.11 | 23.61 |
| Rechter^b^ | C | 251112 | 78.848 | 7 | 2 | 5 | 47.72 | 46.54 | 24.77 |
| Fascho | C | 214 | 0.067 | 7 | 2 | 5 | 68.04 | 67.06 | 25.41 |
| Klimakrise | C | 1447 | 0.454 | 10 | 4 | 5 | 11.39 | 12.61 | 25.72 |
| Rassist | C | 2293 | 0.720 | 9 | 3 | 5 | 57.63 | 63.69 | 27.21 |
| Coronaleugner | C | 29 | 0.009 | 13 | 5 | 4 | 46.94 | 55.87 | 28.41 |
| Transperson | C | 263 | 0.083 | 13 | 4 | 5 | 11.00 | 8.46 | 27.95 |
| Nazi | C | 28368 | 8.907 | 5 | 2 | 5 | 66.22 | 75.25 | 29.46 |
| Sexist | C | 150 | 0.047 | 8 | 3 | 4 | 51.54 | 59.41 | 29.37 |
| Waffenfanatiker | C | 10 | 0.003 | 15 | 6 | 5 | 54.78 | 54.31 | 30.65 |
| Frauenfeind | C | 131 | 0.041 | 12 | 4 | 5 | 56.28 | 63.89 | 33.02 |
| Nationalsozialist | C | 44853 | 14.084 | 19 | 7 | 5 | 43.08 | 61.22 | 32.32 |
| Verschwörungstheoretiker | C | 994 | 0.312 | 24 | 8 | 4 | 57.49 | 57.52 | 33.74 |
| Faschist | C | 5964 | 1.873 | 10 | 3 | 5 | 55.43 | 64.66 | 33.06 |
| Frauenhasser | C | 103 | 0.032 | 12 | 4 | 5 | 59.94 | 68.97 | 35.43 |
| Gendern | C | 248 | 0.078 | 7 | 2 | 5 | 8.31 | 16.17 | 32.08 |
| Klimaaktivist | C | 219 | 0.069 | 15 | 6 | 4 | 13.49 | 16.52 | 34.30 |
| Aluhutträger | C | 2 | 0.001 | 12 | 5 | 5 | 72.18 | 67.40 | 35.77 |
| Feminist | C | 657 | 0.206 | 10 | 4 | 6 | 12.22 | 13.67 | 34.68 |
| Geflüchteter | C | 4728 | 1.485 | 11 | 4 | 4 | 23.16 | 16.18 | 37.64 |
| Atheist | C | 3271 | 1.027 | 9 | 4 | 6 | 3.03 | 8.87 | 40.83 |
| Integration^b^ | C | 115199 | 36.172 | 11 | 4 | 5 | 7.79 | 9.87 | 38.13 |
| Demokratie | C | 142561 | 44.764 | 10 | 4 | 4 | 3.38 | 7.64 | 39.76 |
| Homosexueller | C | 12320 | 3.868 | 12 | 6 | 5 | 15.73 | 12.91 | 39.82 |
| Klimadebatte | C | 177 | 0.056 | 12 | 5 | 4 | 13.17 | 15.28 | 40.23 |
| Erinnerungskultur | C | 7 | 0.002 | 17 | 6 | 4 | 13.04 | 18.35 | 40.56 |
| Nationalist | C | 10035 | 3.151 | 13 | 5 | 6 | 39.78 | 48.03 | 42.17 |
| Waffenbefürworter | C | 7 | 0.002 | 17 | 6 | 4 | 18.78 | 34.57 | 44.49 |
| Erstsprache | C | 548 | 0.172 | 11 | 3 | 5 | 7.66 | 9.51 | 44.31 |
| Linker^b^ | IC | 1714 | 0.538 | 6 | 2 | 5 | 26.63 | 29.64 | 68.40 |
| Flüchtlingswelle | IC | 680 | 0.214 | 17 | 4 | 4 | 47.29 | 45.11 | 68.74 |
| Asylant | IC | 1198 | 0.376 | 7 | 3 | 4 | 53.26 | 48.12 | 69.25 |
| Grünwähler | IC | 34 | 0.011 | 11 | 3 | 5 | 29.47 | 36.68 | 69.41 |
| Gottloser | IC | 17 | 0.005 | 8 | 3 | 5 | 62.46 | 56.67 | 71.17 |
| Heimat | IC | 128074 | 40.215 | 6 | 2 | 4 | 13.12 | 10.71 | 70.76 |
| Asylforderer | IC | 3 | 0.001 | 12 | 5 | 7 | 50.25 | 51.38 | 73.14 |
| Schwuler | IC | 11623 | 3.650 | 7 | 2 | 4 | 59.39 | 54.46 | 73.48 |
| Volk | IC | 342367 | 107.502 | 4 | 1 | 5 | 31.68 | 23.60 | 73.66 |
| Heimatland | IC | 28195 | 8.853 | 10 | 3 | 6 | 18.84 | 17.62 | 77.03 |
| Heimatliebe | IC | 422 | 0.133 | 11 | 4 | 4 | 27.37 | 21.47 | 79.83 |
| Emanze | IC | 172 | 0.054 | 7 | 3 | 4 | 73.43 | 73.09 | 80.30 |
| Transe | IC | 97 | 0.030 | 7 | 2 | 5 | 80.03 | 76.52 | 82.03 |
| Zigeuner | IC | 4747 | 1.491 | 8 | 3 | 5 | 78.01 | 78.38 | 82.58 |
| Sozialschmarotzer | IC | 241 | 0.076 | 17 | 5 | 3 | 82.64 | 75.74 | 83.73 |
| Grünling^b^ | IC | 518 | 0.163 | 9 | 3 | 3 | 66.52 | 57.89 | 82.65 |
| Klimahysterie | IC | 56 | 0.018 | 13 | 5 | 6 | 71.78 | 66.66 | 84.15 |
| Illegaler | IC | 1163 | 0.365 | 8 | 4 | 3 | 78.25 | 75.43 | 84.93 |
| Vaterland | IC | 20468 | 6.427 | 9 | 3 | 4 | 37.17 | 27.07 | 84.93 |
| Schwuchtel | IC | 1058 | 0.332 | 11 | 2 | 5 | 88.04 | 90.36 | 84.89 |
| Kopftuchfrau | IC | 12 | 0.004 | 14 | 4 | 5 | 84.49 | 77.54 | 84.88 |
| Klimawahn | IC | 3 | 0.001 | 9 | 3 | 6 | 69.79 | 69.27 | 85.01 |
| Klimaterrorist | IC | 2 | 0.001 | 16 | 6 | 4 | 75.97 | 74.81 | 85.63 |
| Grünfaschist | IC | 0 | 0.000 | 14 | 4 | 3 | 68.82 | 71.49 | 86.45 |
| Ausländerflut | IC | 23 | 0.007 | 13 | 4 | 4 | 74.31 | 72.21 | 86.46 |
| Ultralinker | IC | 3 | 0.001 | 10 | 4 | 5 | 56.92 | 55.48 | 86.36 |
| Multikultiwahn | IC | 3 | 0.001 | 14 | 5 | 6 | 75.11 | 71.30 | 86.90 |
| Linksfaschist | IC | 37 | 0.012 | 15 | 4 | 4 | 69.00 | 71.77 | 86.75 |
| Nigger | IC | 2018 | 0.634 | 6 | 2 | 3 | 96.92 | 92.80 | 88.69 |
| Neger | IC | 4112 | 1.291 | 5 | 2 | 3 | 93.65 | 88.67 | 90.55 |

^a^Data from the survey by Hons et al. (2026)

^b^Target words with ambiguous meanings and usage in the corpus. Not subjected to the analysis of word characteristics.
