# Supplementary material for: The neural dynamics of political socio-pragmatic violations: an ERP study
Source: Front Hum Neurosci. 2026 Jun 29;20:1820376. doi: 10.3389/fnhum.2026.1820376 (PMC13357823; doi:10.3389/fnhum.2026.1820376)
Supplement: Supplementary file 3 [file Table_3.DOCX]

**Supplementary Table S3.** N400 linear mixed effects model results with a fixed time window ranging from 300 ms to 500 ms post-stimulus onset.

| Variable | *b* | *SE* | *df* | *t* | *p* |
| --- | --- | --- | --- | --- | --- |
| Coherence | -0.001 | 0.024 | 61.419 | -0.052 | .959 |
| Quotation | -0.141 | 0.013 | 788.960 | -11.113 | < .001 |
| Pejorative Weight | -0.014 | 0.020 | 62.394 | -0.728 | .469 |
| Coherence*Quotation | 0.032 | 0.013 | 787.343 | 2.518 | .012 |
| Coherence*Pej. Weight | -0.014 | 0.023 | 59.730 | -0.616 | .540 |
| Quotation*Pej. Weight | 0.022 | 0.016 | 51.807 | 1.365 | .178 |
| Coherence*Quotation*Pej. Weight | -0.019 | 0.019 | 50.675 | -0.988 | .328 |
