# Supplementary material for: The neural dynamics of political socio-pragmatic violations: an ERP study
Source: Front Hum Neurosci. 2026 Jun 29;20:1820376. doi: 10.3389/fnhum.2026.1820376 (PMC13357823; doi:10.3389/fnhum.2026.1820376)
Supplement: Supplementary file 4 [file Table_4.DOCX]

**Supplementary Table S4.** Post-hoc contrasts of the Coherence*Quotation interaction of the N400 linear mixed effects model with a fixed time window ranging from 300 ms to 500 ms post-onset.

| Contrast | Estimate | *SE* | *df* | *z-ratio* | *p* |
| --- | --- | --- | --- | --- | --- |
| C-NoQM – IC-NoQM | 0.061 | 0.055 | Inf | 1.127 | .449 |
| C-QM – IC-QM | -0.066 | 0.055 | Inf | -1.215 | .449 |
| IC-NoQM – IC-QM | -0.345 | 0.036 | Inf | -9.624 | < .001 |
| C-NoQM – C-QM | -0.217 | 0.036 | Inf | -6.075 | < .001 |
