# Supplementary material for: The neural dynamics of political socio-pragmatic violations: an ERP study
Source: Front Hum Neurosci. 2026 Jun 29;20:1820376. doi: 10.3389/fnhum.2026.1820376 (PMC13357823; doi:10.3389/fnhum.2026.1820376)
Supplement: Supplementary file 5 [file Table_5.DOCX]

**Supplementary Table S5.** N400 linear mixed effects model results with mean SDO-7 scores as subject-wise random effects variable.

| Variable | *b* | *SE* | *df* | *t* | *p* |
| --- | --- | --- | --- | --- | --- |
| Coherence | 0.001 | 0.024 | 60.926 | 0.030 | .976 |
| Quotation | -0.105 | 0.013 | 711.364 | -8.174 | < .001 |
| Pejorative Weight | -0.037 | 0.021 | 33.030 | -1.817 | .078 |
| Coherence*Quotation | 0.073 | 0.013 | 707.164 | 5.697 | < .001 |
| Coherence*Pej.Value | -0.014 | 0.026 | 28.985 | -0.541 | .592 |
| Quotation*Pej.Value | 0.018 | 0.019 | 27.346 | 0.953 | .349 |
| Coherence*Quotation*Pej.Value | 0.005 | 0.026 | 20.923 | 0.213 | .833 |
